# Hypertension, kidney disease, HIV and antiretroviral therapy among Tanzanian adults: a cross-sectional study

**DOI:** 10.1186/s12916-014-0125-2

**Published:** 2014-07-29

**Authors:** Robert N Peck, Rehema Shedafa, Samuel Kalluvya, Jennifer A Downs, Jim Todd, Manikkam Suthanthiran, Daniel W Fitzgerald, Johannes B Kataraihya

**Affiliations:** Department of Internal Medicine, Catholic University of Health and Allied Sciences, PO Box 5034 Mwanza, Tanzania; Department of Internal Medicine, Bugando Medical Centre, Mwanza, Tanzania; Center for Global Health, Weill Cornell Medical College, New York, USA; Population Health Department, London School of Hygiene & Tropical Medicine, London, UK; Division of Nephrology and Hypertension, Weill Cornell Medical College, New York, USA

**Keywords:** Hypertension, Blood pressure, HIV, Antiretroviral therapy, Sub-Saharan Africa, Kidney disease

## Abstract

**Background:**

The epidemics of HIV and hypertension are converging in sub-Saharan Africa. Due to antiretroviral therapy (ART), more HIV-infected adults are living longer and gaining weight, putting them at greater risk for hypertension and kidney disease. The relationship between hypertension, kidney disease and long-term ART among African adults, though, remains poorly defined. Therefore, we determined the prevalences of hypertension and kidney disease in HIV-infected adults (ART-naive and on ART >2 years) compared to HIV-negative adults. We hypothesized that there would be a higher hypertension prevalence among HIV-infected adults on ART, even after adjusting for age and adiposity.

**Methods:**

In this cross-sectional study conducted between October 2012 and April 2013, consecutive adults (>18 years old) attending an HIV clinic in Tanzania were enrolled in three groups: 1) HIV-negative controls, 2) HIV-infected, ART-naive, and 3) HIV-infected on ART for >2 years. The main study outcomes were hypertension and kidney disease (both defined by international guidelines). We compared hypertension prevalence between each HIV group versus the control group by Fisher’s exact test. Logistic regression was used to determine if differences in hypertension prevalence were fully explained by confounding.

**Results:**

Among HIV-negative adults, 25/153 (16.3%) had hypertension (similar to recent community survey data). HIV-infected adults on ART had a higher prevalence of hypertension (43/150 (28.7%), *P* = 0.01) and a higher odds of hypertension even after adjustment (odds ratio (OR) = 2.19 (1.18 to 4.05), *P* = 0.01 in the best model). HIV-infected, ART-naive adults had a lower prevalence of hypertension (8/151 (5.3%), *P* = 0.003) and a lower odds of hypertension after adjustment (OR = 0.35 (0.15 to 0.84), *P* = 0.02 in the best model). Awareness of hypertension was ≤25% among hypertensive adults in all three groups. Kidney disease was common in all three groups (25.6% to 41.3%) and strongly associated with hypertension (*P* <0.001 for trend); among hypertensive participants, 50/76 (65.8%) had microalbuminuria and 20/76 (26.3%) had an estimated glomerular filtration rate (eGFR) <60 versus 33/184 (17.9%) and 16/184 (8.7%) participants with normal blood pressure.

**Conclusions:**

HIV-infected adults on ART >2 years had two-fold greater odds of hypertension than HIV-negative controls. HIV-infected adults with hypertension were rarely aware of their diagnosis but often have evidence of kidney disease. Intensive hypertension screening and education are needed in HIV-clinics in sub-Saharan Africa. Further studies should determine if chronic, dysregulated inflammation may accelerate hypertension in this population.

## Background

High blood pressure is the leading risk factor for disease worldwide and accounts for 7% of global disability-adjusted life years (DALYs) and nearly 10 million deaths per year [1]. Despite global declines in blood pressure, the blood pressures of adults in sub-Saharan Africa (SSA) continue to rise [2,3], and the age-adjusted prevalence of hypertension in SSA is estimated to be the highest of any region in the world [4,5].

HIV remains common in SSA where 69% of HIV-infected persons reside and one in every twenty adults is infected [6]. With half of the eligible HIV-infected persons in SSA on antiretroviral therapy (ART) as of 2010 [7], the infection-related mortality rates have begun to decline and life expectancy has increased [8], which will likely mean more cardiovascular disease mortality among HIV-infected adults as already seen in developed countries [9,10]. On a population level, in regions with high HIV prevalence, ART-related weight gain among large numbers of HIV-infected adults could lead to an ‘unmasking’ of an epidemic of hypertension and an overall increase in the prevalence of cardiovascular diseases [11].

The impact of HIV and ART on hypertension remains controversial. A recent systematic review and meta-analysis found that HIV-infected adults in SSA generally have lower blood pressures than uninfected adults [12], and a large, population-based study from South Africa showed that hypertension (particulary stage 2 hypertension) was less common among HIV-infected adults [13], but both studies noted that data were lacking for HIV-infected adults on long-term ART in SSA. ART could lead to hypertension due to weight gain, ART drug toxicities or through an immune-related phenomenon. In the US and Europe, some studies have confirmed higher rates of hypertension among HIV-infected adults on ART compared to uninfected adults [14], but most studies have shown no difference [12,15-18]. In two recent studies from SSA, the prevalence of hypertension among HIV-infected adults was high but, since only HIV-infected adults were enrolled, it remains unclear whether this was due to HIV infection, ART or simply a high community-wide prevalence of hypertension [19,20]. In addition, although our prior work demonstrated that kidney disease is common among HIV-infected adults in our region [21,22], the relationship between hypertension and kidney disease (which can be a complication of hypertension, a cause of secondary hypertension or a complication of HIV or ART) remains unknown.

In view of the existing gap in knowledge, we conducted this prospective study to assess the prevalence of hypertension and kidney disease among: 1) HIV-infected, ART-naive adults, 2) HIV-infected adults on ART for >2 years and 3) HIV-negative adults (controls) drawn from the same population. We hypothesized that, compared to HIV-negative controls, hypertension would be more common among HIV-infected adults on ART (even after adjusting for confounders) and would be frequently associated with kidney disease.

## Methods

### Study design

This was a cross-sectional study designed to compare the prevalence of hypertension between HIV-negative adults and two groups of HIV-infected adults.

### Study area

The study was conducted in the outpatient HIV clinic of the Bugando Medical Centre (BMC) in Mwanza, Tanzania. BMC is the zonal hospital for the Lake Victoria Zone in northwest Tanzania, serving a population of approximately 13 million. The HIV prevalence in the Lake Zone is 6% [23], similar to the national average. The BMC HIV clinic provides care to 3,500 patients of whom 2,700 are currently on ART. Patients are referred to BMC from surrounding community-based voluntary counseling and testing centers in the city of Mwanza. According to Tanzanian national guidelines, all HIV-infected patients must be assigned a treatment partner who is typically a family member, friend or partner.

HIV-infected patients fulfilling Tanzanian national criteria for ART are started on treatment and are seen monthly or bi-monthly at the BMC clinic. At the time of the study, Tanzanian criteria for starting ART included World Health Organization (WHO) Clinical Stage III disease with CD4 count <350 cells/μl, Stage IV disease regardless of CD4 count, or CD4 count <200 cells/μl. The first-line ART regimen consisted of either tenofovir/emcitrabine or zidovudine/lamivudine + nevirapine or efavirenz. Protease inhibitors (PIs) were only given as second-line ART, in accordance with Tanzanian national guidelines [24].

### Study population

Three study groups of non-pregnant adults (>18 years old) were recruited from the BMC HIV clinic:

1. HIV-negative adult treatment partners (the control group),

2. HIV-infected adults enrolled in the last three months and not yet on ART (the HIV-infected, ART-naive group), and

3. HIV-infected adults on ART for >2 years (the HIV-infected, on ART group).

Consecutive HIV-infected adults who met the criteria for study groups 2 or 3 were asked to participate and, if they agreed, their treatment partners were asked to participate in the HIV-negative control group in order to provide a control population with similar socioeconomic status to the two groups of HIV-infected adults. Pregnant women were not eligible for the study. Adults who failed to attend a follow-up visit on the day after enrollment were excluded.

### Study procedure

On the day of enrollment, the WHO STEPS questionnaire was administered by the study investigators to determine the prevalence of hypertension and hypertension risk factors [25]. The WHO STEPS questionnaire includes questions regarding prior hypertension testing/diagnosis/treatment, other non-communicable disease and standard protocols for physical examination (as described below). Additional questions were added regarding HIV diagnosis and treatment.

After completing the questionnaire, we conducted a physical examination, including weight and height to assess body mass index (BMI) and waist and hip circumferences. We measured weight to the nearest 0.1 kg using a digital Seca® 813 weight measuring scale (Seca, Hamburg, Germany) with participants wearing minimal clothing and shoes removed. Height was measured to the nearest 0.1 cm using a Seca® 213 stadiometer. Waist and hip circumferences were measured twice to the nearest 0.1 cm using a 203 cm Seca® measuring tape. For each of these measurements, the mean of the two values was used.

Blood pressure was measured at least three times over the course of two days by a registered nurse or doctor using a mercury sphygmomanometer. All blood pressure measurements were taken after five minutes of resting quietly and study subjects sat with arm supported at the level of the heart. On the day of enrollment, in accordance with the WHO STEPS protocol [25], at least two measurements were taken one minute apart on alternating arms. If there was >10 mmHg difference in either the systolic and/or diastolic readings, comparing these two readings, further readings were taken until two consecutive measurements were concordant within this range. Average systolic and diastolic pressures were calculated from the last two readings. An additional blood pressure measurement was made on the following day using the same procedure.

### Laboratory analysis

At the time of enrollment, venous blood and a clean-catch urine sample were obtained. The CD4 T-cell count was measured using an automated BD FACS Calibur Machine (BD Biosciences, San Jose, CA, USA). A serum creatinine level was measured using Cobas Integra 400 Plus Analyzer (Roche Diagnostic Limited, Basel, Switzerland). An estimated glomerular filtration rate (eGFR) was calculated using the Chronic Kidney Disease Epidemiology Collaboration (CKD-EPI) equation (without ethnic factor) since this equation is recommended by internationally-recognized Kidney Disease Improving Global Outcomes (KDIGO) guidelines and has been shown to be the most accurate eGFR equation for African adults [26-28]. Urine samples were tested for microalbuminuria using Micral B Test Strips (Roche, Mannheim, Germany) as used in our prior studies [21,29]. In order to maximize our specificity, we defined microalbuminuria as a urine albumin concentration of >50 mg/L [30]. In women whose last menstrual period was >1 month prior to the date of study interview, a urine pregnancy test was performed.

### Definitions

The primary outcome of this study was hypertension. Hypertension was defined as a sustained elevation of systolic blood pressure (SBP) ≥140 mmHg and/or diastolic blood pressure (DBP) ≥90 mmHg on two different days or current antihypertensive therapy, according to the Joint National Committee 7 (JNC-7) definition [31]. The grade of hypertension was also defined according to JNC-7 using the average of three blood pressure readings: normal is SBP <120 mmHg and DBP <80 mmHg, prehypertension is SBP 120 to 139 mm Hg or DBP 80 to 89 mm Hg, Stage I hypertension is SBP 140 to 159 mm Hg or DBP 90 to 99 mm Hg and Stage II hypertension is SBP >160 mm Hg or DBP >100 mm Hg.

Central obesity was defined as a waist-hip ratio of ≥0.85 for women and waist-hip ratio ≥0.90 for men according to the WHO [25]. Chronic Kidney Disease (CKD) was defined as eGFR <60 mL/minute and/or microalbuminuria according to KDIGO [26].

### Statistical analysis

The primary outcome of the study was hypertension (as defined above). The primary study analysis was to compare the prevalence of hypertension between each HIV-infected group and the HIV-negative control group. According to a recent population-based survey, 17% of adults in Mwanza city have hypertension (Kavishe BB, Mwanza Interventional Trials Unit, personal communication) and we hypothesized that 30% of HIV-infected adults on ART would have diabetes mellitus. Using Fishers exact test, we calculated that 150 patients in each group would provide 80% power to detect this difference for *P* <0.05.

Data analysis was done using STATA version 11 (San Antonio, TX, USA). Descriptive statistics were computed by determining median [interquartile range] for continuous variables and proportions (percentages) for categorical variables. Differences between medians were determined using the rank sum test and differences between proportions were determined using Fisher’s exact test. For ordered categorical variables, the nonparametric test for trend was used. *P*-values of less than 0.05 were considered significant.

Multiple logistic regression models were performed in order to determine whether the relationship between HIV status and hypertension could be explained by confounding. All baseline characteristics, including past or current exposure to individual ART drugs, were evaluated by a pre-determined, minimally adjusted logistic regression model adjusting for age and sex (since these were expected to differ between groups). Additional, pre-determined multivariable analyses were performed to adjust for BMI and waist-hip ratio (since these were the factors most highly expected to explain differences in hypertension prevalence between groups) as well as fully-adjusted models including all variables with a *P*-value of <0.05 by minimally adjusted multivariate analysis. BMI and waist-hip ratio were not included together in any models due to collinearity. Variables associated with HIV infection and ART use were not included in the multivariable models due to collinearity with the group variables and the smaller number of subjects with these additional variables. For associated factors, odds ratios (OR) and 95% confidence intervals (95% CI) were determined. The likelihood ratio test was used to compare the logistic regression models. We also performed multivariable linear regression to determine factors associated with increased SBP and DBP, including all of the same variables that were included in the best-fit multivariable logistic regression model.

### Ethical issues

The study was approved by the Institutional Review Boards at BMC and Weill Cornell Medical College. All study participants were informed about the study by a nurse or doctor fluent in Kiswahili and provided written informed consent before participation. All results were made available to clinicians and recorded in the patient’s file. Disease management was conducted by the health care workers of the HIV clinic according to BMC and Tanzanian management protocols.

## Results

### Enrollment

Between October 2012 and April 2013, 488 consecutive adults were screened. Seven were pregnant (three HIV-infected ART-naive and four HIV-infected on ART), leaving 481 eligible adults. A total of 454/481 (94%) of eligible adults were enrolled: 153 HIV-negative adults (controls), 151 HIV-infected ART-naive adults and 150 HIV-infected adults on ART. Twenty-seven were excluded from the study because they did not return for follow-up testing (eleven HIV-negative controls, nine HIV-infected ART-naive, seven HIV-infected on ART).

### Baseline characteristics

Table 1 is a summary of the baseline characteristics of the three groups.

**Table 1:** **Baseline characteristics of the 454 Tanzanian adult study participants**

**Variable**	**HIV-negative**	**HIV-infected,**	**HIV-infected,**
**Proportion (%)**	**control**	**ART-naive**	**on ART**
**Median [IQR]**	**(number = 153)**	**(number = 151)**	**(number = 150)**
**Female**	94 (61.4%)	89 (58.9%)	115 (76.7%)
**Age (in years)**	38 [32 to 46]	37 [32 to 44]	40 [38 to 47]
**Education level**			
Incomplete primary	27 (17.7%)	25 (16.6%)	30 (20.0%)
Complete primary	82 (53.6%)	98 (64.9%)	94 (62.7%)
Secondary and above	44 (28.8%)	28 (18.5%)	26 (17.3%)
**Work type**			
Manual	109 (71.2%)	114 (75.5%)	105 (70.0%)
Office	44 (28.8%)	37 (24.5%)	45 (30.0%)
**Vigorous work**	15 (9.8%)	21 (13.9%)	33 (22.0%)
**Mode of transportation**			
Walking or bicycle	130 (85.0%)	110 (72.9%)	117 (78.0%)
Motorized vehicle	23 (15.0%)	41 (27.1%)	33 (22.0%)
**Ease of Living Index** ^**a**^			
Low	86 (56.2%)	94 (62.3%)	81 (54.0%)
Middle	20 (13.1%)	24 (15.9%)	26 (17.3%)
Higher	47 (30.7%)	33 (21.9%)	43 (28.7%)
**Ever smoking**	6 (3.9%)	16 (10.6%)	10 (6.7%)
**Current smoker**	5 (3.3%)	4 (2.7%)	0
**Fruit and vegetables servings/week**	6 [4-12]	8 [5-12]	9 [6 to 13]
**Sugary drinks/day**	1 [0.5 to 2]	1 [0.5 to 2]	1 [1 to 2]
**Current alcohol use**			
None	119 (77.8%)	120 (79.5%)	136 (90.7%)
< once/week	19 (12.4%)	16 (10.6%)	8 (5.3%)
≥ once/week	15 (9.8%)	15 (9.9%)	6 (4.0%)
**Body Mass Index (BMI) in kg/m** ^**2**^	23.8 [22.3 to 25.8]	22 [20.2 to 24.3]	23.7 [21.5 to 27.9]
BMI <18.5	5 (3.3%)	18 (11.9%)	4 (2.7%)
BMI 18.5 to 25	104 (68.0%)	113 (74.8%)	80 (53.3%)
BMI 25 to 30	33 (21.6%)	9 (6.0%)	45 (30.0%)
BMI >30	11 (7.2%)	11 (7.3%)	21 (14.0%)
**Waist-hip ratio**	0.84 [0.82 to 0.87]	0.84 [0.80 to 0.89]	0.87 [0.82 to 0.91]
**Central obesity** ^**b**^	44 (29.1%)	56 (37.1%)	78 (52.0%)
**Diabetes mellitus**	0	1 (0.7%)	1 (0.7%)
**Current CD4 T-cell count in cells/μL**	NA	215 [150 to 321]	378 [263 to 521]
**Nadir CD4 T-cell count in cells/μL**	NA	209 [120 to 302]	118.5 [68 to 196]
**ART duration (months)**	NA	NA	56 [31 to 68]
**Protease inhibitor use**	NA	NA	18 (12.0%)

^a^Ease of Living Index was defined according to the presence of water, electricity and/or flushing toilets inside the home. Low = 0/3, Medium = 1 to 2/3, Higher = 3/3. ^b^Defined as waist-hip ratio of ≥0.85 for women and waist/hip ratio ≥0.90 for men. ART, antiretroviral therapy; IQR, interquartile range.

The characteristics of the three groups were broadly similar. Notable differences included that HIV-infected adults on ART were slighly older (median age 40 [38 to 47] years versus 38 [32 to 46] years and 37 [32 to 44] years in the other two groups), more female (76.7% versus 61.4% and 58.9% in the other two groups) and had a higher prevalence of central obesity (52.0% versus 29.1% and 37.1%). HIV-infected, ART-naive adults, on the other hand, had lower mean BMI (22.0 [20.2 to 24.3] kg/m^2^ versus 23.8 [22.3 to 25.8] and 23.7 [21.5 to 27.9]) and were severely immunosuppressed (mean CD4 T-cell count 215 [150 to 321] cells/ul versus 378 [263 to 521] in the group on ART).

### Hypertension outcomes

Table 2 shows hypertension outcomes in the three groups. The prevalence of hypertension in the 153 HIV-negative controls was 25/153 (16.3%). The prevalence of hypertension was lowest in the 151 HIV-infected, ART-naive group (8/151 (5.3%), *P* = 0.003) and highest in the 150 HIV-infected adults on ART >2 years (43/150 (28.7%), *P* = 0.01). The median SBP and DBP were both lower in the HIV-infected, ART-naive group (*P* = 0.007 and *P* = 0.04, respectively). The grade of hypertension was also higher among the HIV-infected adults on ART versus controls (*P* = 0.01 for trend).

**Table 2:** **Hypertension outcomes among the 454 Tanzanian adult study participants**

**Variable**	**HIV-negative**	**HIV-infected,**	***P*** **-value**	**HIV-infected,**	***P*** **-value**
**Proportion (%)**	**control**	**ART-naïve**	**versus**	**on ART**	**versus**
**Median [IQR]**	**(number = 153)**	**(number = 151)**	**control**	**(number = 150)**	**control**
**Hypertension** ^**a**^	25 (16.3%)	8 (5.3%)	**0.003** ^**b**^	43 (28.7%)	**0.01**
**Systolic blood pressure**	120 [110 to 130]	115 [110 to 120]	**0.007**	120 [110 to 137.5]	0.16
**Diastolic blood pressure**	75 [70 to 80]	75 [70 to 80]	**0.04**	80 [70 to 90]	0.11
**Heart rate**	73 [70 to 77]	74 [70 to 79]	0.35	74 [69.5 to 85]	0.18
**Hypertension grade**			*P*-value for trend		*P*-value for trend
Normal	66 (43.1%)	69 (45.7%)		49 (32.7%)	
Prehypertension	62 (40.5%)	74 (49.0%)	0.16	58 (38.7%)	**0.01**
Grade 1 hypertension	20 (13.1%)	7 (4.6%)		33 (22.0%)	
Grade 2 hypertension	5 (3.3%)	1 (0.7%)		10 (6.7%)	

^a^Primary outcome: hypertension defined as average SBP >140 and/or DBP >90 and/or currently taking antihypertensive medications. ^b^Throughout the tables, bolding has been used to indicate those p-values and odds ratios which were statistically significant. ART, antiretroviral therapy; IQR, interquartile range.

Rates of hypertension awareness ranged from 3/25 (12%) and 1/8 (12.5%) in the control and HIV-infected, ART-naive group to 11/43 (25.6%) in the HIV-infected on ART group. Rates of current hypertension treatment ranged from none in the control and HIV-infected, ART-naive groups to 7/43 (16.3%) in the HIV-infected on ART group. Rates of hypertension control ranged from none in the control and HIV-infected, ART naive groups to 1/43 (2.3%) in the HIV-infected on ART group. Among HIV-negative controls, 86/153 (56.2%) reported never having had their blood pressure checked and only 40/153 (26.1%) reported having their blood pressure checked within the last year. Rates of prior blood pressure testing were similar in HIV-infected, ART-naive adults (83/151 (55.0%) never checked and 41/151 (27.2%) checked in last year, *P* = 0.82) but slightly higher among HIV-infected adults on ART (64/150 (42.7%) and 47/150 (31.3%) respectively, *P* = 0.04).

### Factors associated with hypertension

Table 3 shows the factors associated with hypertension by predetermined, partially adjusted multivariable analysis (adjusted for age and sex). As shown, age (OR = 1.07 [1.04 to 1.09]), vigorous work (OR = 0.33 [0.13 to 0.88]), current alcohol use ≥ once/week (OR = 0.13 [0.02 to 0.99]), and BMI (OR = 1.09 [1.03 to 1.15]) were all associated with hypertension. Current CD4 T-cell count (OR = 4.33 [1.51 to 12.40] for CD4 T-cell count >500cells/μL versus <200cells/μL) was also significantly associated with hypertension. Of note, the current CD4 T-cell count was also associated with both SBP and DBP by linear regression (β = 0.022 [0.014 to 0.029], *P* <0.001 and β = 0.011 [0.006 to 0.017], *P* <0.001, respectively).

**Table 3:** **Factors associated with hypertension among 454 Tanzanian adults by multivariable logistic regression adjusted for age and sex**

**Variable**	**No hypertension**	**Hypertension**	**Odds ratio [95% CI]**
**Proportion (%)**	**(number = 378)**	**(number = 76)**	
**Median [IQR]**			
**Female**	251 (66.4%)	47 (61.8%)	1.10 [0.64 to 1.91]
**Age (in years)**	38 [32 to 44]	45 [39.5 to 52]	**1.07 [1.04 to 1.09]**
**Education level**			
Incomplete primary	72 (19.1%)	10 (13.2%)	1
Complete primary	221 (58.5%)	53 (69.7%)	2.09 [0.97 to 4.50]
Secondary and above	85 (22.5%)	13 (82.9%)	1.57 [0.61 to 4.02]
**Work type**			
Manual	274 (72.5%)	54 (71.1%)	1
Office	104 (27.5%)	22 (29.0%)	1.08 [0.62 to 1.91
**Vigorous work**	64 (16.9%)	5 (6.6%)	**0.33 [0.13 to 0.88]**
**Transportation**			
Walking or bicycle	301 (79.6%)	56 (74.7%)	1
Motorized vehicle	77 (20.4%)	20 (26.3%)	1.32 [0.73 to 2.39]
**Ease of Living Index**			
Low	219 (57.9%)	42 (55.3%)	1
Middle	54 (14.3%)	16 (21.1%)	1.96 [0.99 to 3.91]
Higher	105 (27.8%)	18 (23.7%)	0.96 [0.52 to 1.79]
**Ever smoking**	26 (6.9%)	6 (7.9%)	1.02 [0.37 to 2.76]
**Current smoker**	7 (1.9%)	2 (2.6%)	1.41 [0.25 to 8.14]
**Fruit and vegetables servings/week**	8 [5 to 12]	9 [5 to 13]	1.02 [0.98 to 1.06]
**Sugary drinks/day**	1 [1,2]	1 [0.5 to 2]	1.17 [0.98 to 1.40]
**Current alcohol**			
None	307 (81.2%)	68 (89.5%)	1
< once/Week	36 (9.5%)	7 (9.2%)	0.77 [0.31 to 1.89]
≥ once/Week	35 (9.3%)	1 (1.3%)	**0.13 [0.02 to 0.99]**
**Body Mass Index (BMI) in kg/m** ^**2**^	22.9 [20.9 to 25.1]	24.2 [22.3 to 28.2]	**1.09 [1.03 to 1.15]**
BMI <18.5	25 (6.6%)	2 (2.6%)	1
BMI 18.5 to 25	255 (67.5%)	42 (55.3%)	1.53 [0.34 to 6.84]
BMI 25 to 30	67 (17.7%)	20 (26.3%)	3.39 [0.73 to 15.77]
BMI >30	31 (8.2%)	12 (15.8%)	3.40 [0.67 to 17.15]
**Waist-to-Hip Ratio**	0.84 [0.81 to 0.89]	0.86 [0.83 to 0.91]	1.03 [0.98 to 1.08] for each 0.01 increase
**Central obesity** ^**a**^	142 (37.6%)	36 (47.4%)	1.54 [0.90 to 2.64]
**Current CD4 T-cell** **count (in cells/μL)** ^**b**^	267.5 [180 to 385]	453 [323 to 590]	**1.003 [1.002 to 1.005]** **for each cell/μL**
<200	71 (28.4%)	6 (11.8%)	1
200 to 350	98 (39.2%)	10 (19.6%)	0.88 [0.30 to 2.63]
350 to 500	46 (18.4%)	15 (29.4%)	2.44 [0.85 to 7.01]
>500	35 (14.0%)	20 (39.2%)	**4.33 [1.51 to 12.40]**

^a^Defined as waist/hip ratio of ≥0.85 for women and waist/hip ratio ≥0.90 for men. ^b^For all 301 study subjects with HIV including 50 with hypertension and 250 without hypertension. CI, confidence interval; IQR, interquartile range.

Among variables that were only available for HIV-infected adults on ART, only the use of protease inhibitors (OR = 3.14 [1.10 to 8.98]) was significantly associated with hypertension by logistic regression adjusted for age and sex. The following variables were not significantly associated with hypertension: duration of ART (OR = 1.017 [0.999 to 1.034]), zidovudine use (OR = 0.81 [0.39 to 1.70]), stavudine use (OR = 0.97 [0.46 to 2.04]), tenofovir use (OR = 1.26 [0.59 to 2.70]), efavirenz use (OR = 0.84 [0.39 to 1.75]), and nevirapine use (OR = 1.13 [0.51 to 2.51]).

Table 4 displays the multivariable models used to estimate the impact of HIV and ART status on hypertension status. HIV-infected adults on ART had a significantly higher risk of hypertension than HIV-negative controls, even after adjusting for differences in age and sex (OR = 2.13 [1.18 to 3.85]). Further adjustment for BMI, waist-to-hip ratio, vigorous work and alcohol use did not change these estimates. In contrast, HIV-infected, ART-naive adults had a significantly lower risk of hypertension, even after adjustment for difference in age and sex (OR = 0.32 [0.14 to 0.75]). Further adjustment for BMI, waist-to-hip ratio, vigorous work and alcohol use did not change these estimates. The likelihood ratio test showed that the model with HIV and ART status, along with age, sex, BMI, vigorous work and alcohol use, best explained the differences in hypertension in this study. By linear regression, adjusting for age, sex, BMI, vigorous work and alcohol use, the HIV-infected, ART-naive group had lower SBP and DBP, but this was only statistically significant for SBP (β = −3.84 [−6.89 to −0.79], *P* = 0.01 and β = −1.96 [−4.09 to 0.17], *P* = 0.07 respectively). The HIV-infected on ART group had higher SBP and DBP, but this was not statistically significant for either (β = 1.32 [−1.85 to 4.48], *P* = 0.41 and β = 1.27 [−0.93 to 3.48], *P* = 0.26, respectively).

**Table 4:** **Multivariable logistic regression models for association between HIV status and hypertension to assess for confounding**

**Model**	**Controls (number = 153)**	**HIV-infected, ART naive**	***P*** **-value versus control**	**HIV-infected, on ART**	***P*** **-value versus control**	**Likelihood ratio test (compared to unadjusted model)**
		**(number = 151)**		**(number = 150)**		
**1) Unadjusted**	1	**0.28 [0.12 to 0.66]**	**0.003**	**2.06 [1.18 to 3.59]**	**0.01**	0
**2) Adjusted for age + sex**	1	**0.32 [0.14 to 0.75]**	**0.009**	**2.13 [1.18 to 3.85]**	**0.01**	**21.7, 2 d.f.**
**3) Adjusted for age + sex + body mass index (BMI)**	1	**0.34 [0.14 to 0.79]**	**0.01**	**2.04 [1.12 to 3.67]**	**0.02**	**25.7, 3 d.f.**
**4) Adjusted for age + sex + waist-hip ratio (WHR)**	1	**0.31 [0.13 to 0.73]**	**0.007**	**2.04 [1.12 to 3.71]**	**0.02**	**22.2, 3 d.f.**
**5) Adjusted for age + sex + BMI + vigorous work + alcohol**	1	**0.35 [0.15 to 0.84]**	**0.02**	**2.19 [1.18 to 4.05]**	**0.01**	**37.7, 5 d.f.** ^**a**^
**6) Adjusted for age + sex + WHR + vigorous work + alcohol**	1	**0.32 [0.14 to 0.77]**	**0.01**	**2.19 [1.17 to 4.10]**	**0.01**	**35.4, 5 d.f.**

^a^Best fit model. All models are comparing to HIV-negative controls. Models 1, 2, 3 and 4 were predetermined based on most likely confounders. Models 5 + 6 included other baseline characteristics significantly associated with hypertension in the minimally-adjusted model. ART, antiretroviral therapy.

### Renal disease outcomes

Table 5 displays the renal disease outcomes among the 454 study participants. The overall prevalence of chronic kidney disease among the 153 HIV-negative controls was 25.6%. Among the 150 HIV-infected adults on ART >2 years, the prevalence of chronic kidney disease was 41.3% (*P* = 0.004 versus the control group) and microalbuminuria was also more common than among controls (58/150 (38.7%) versus 31/153 (20.3%), *P* = 0.001). None of the commonly used antiretrovirals (ARVs) were significantly associated with chronic kidney disease by Fisher’s exact test (*P* = 0.73 for tenofovir, *P* = 0.87 for zidovudine, *P* = 0.40 for stavudine, *P* = 1.00 for nevirapine, *P* = 1.00 for efavirenz, *P* = 0.08 for protease inhibitors,).

**Table 5:** **Kidney disease outcomes among the 454 Tanzanian adult study participants**

**Variable**	**HIV-negative controls**	**HIV-infected, ART-naive**	***P*** **-value versus control**	**HIV-infected, on ART**	***P*** **-value versus control**
**Proportion (%)**					
**Median [IQR]**	**(number = 153)**	**(number = 151)**		**(number = 150)**	
**Kidney disease** ^**a**^	39 (25.6%)	52 (34.4%)	0.09	62 (41.3%)	**0.004**
**Creatinine (mmol/L)**	68.3 [55.6 to 81.1]	70.9 [57.9 to 89]	0.49	64.3 [54.7 to 81.8]	0.69
**eGFR**	106.5 [78.8 to 119.3]	104.9 [79.9 to 116.7]	0.62	104.4 [82.1 to 115.6]	0.67
**eGFR <60 mL/min**	20 (13.1%)	19 (12.6%)	0.89	16 (10.7%)	0.52
**Microalbuminuria**	31 (20.3%)	43 (28.5%)	0.10	58 (38.7%)	**0.001**

^a^The primary kidney disease outcome: defined as microalbuminuria and/or eGFR <60 mL/min. ART, antiretroviral therapy; eGFR, estimated glomerular filtration rate; IQR, interquartile ratio.

Table 6 shows the association between kidney disease and grade of hypertension both overall and in each of the three study groups. Overall, higher grades of hypertension were associated with higher rates of kidney disease, microalbuminuria and eGFR <60 (*P* <0.0001 for trend for all three variables). Similar trends were seen in all three study groups.

**Table 6:** **Association between kidney disease and grade of hypertension among all 454 Tanzanian adult study participants and in each of the three study groups**

**Among 454 Tanzanian adult study participants**					
	**Normal**	**Prehypertension**	**Hypertension Grade 1**	**Hypertension Grade 2**	***P*** **-value for trend**
	**(number = 184)**	**(number = 194)**	**(number = 60)**	**(number = 16)**	
**Kidney Disease** ^**a**^	44 (23.9%)	58 (29.9%)	40 (66.7%)	11 (68.8%)	**<0.001**
**Microalbuminuria**	33 (17.9%)	49 (25.3%)	40 (66.7%)	10 (62.5%)	**<0.001**
**eGFR <60**	16 (8.7%)	19 (9.8%)	14 (23.3%)	6 (37.5%)	**<0.001**
**Among 153 HIV-negative adults (controls)**					
	**Normal**	**Prehypertension**	**Hypertension Grade 1**	**Hypertension Grade 2**	**P-value for trend**
	**(number = 66)**	**(number = 62)**	**(number = 20)**	**(number = 5)**	
**Kidney Disease**	8 (12.1%)	15 (24.2%)	13 (65.0%)	3 (60.0%)	**<0.001**
**Microalbuminuria**	4 (6.1%)	12 (19.4%)	13 (65.0%)	2 (40.0%)	**<0.001**
**eGFR <60**	5 (7.6%)	6 (9.7%)	6 (30.0%)	3 (60.0%)	**<0.001**
**Among 151 HIV-infected, ART-naive adults**					
	**Normal**	**Prehypertension**	**Hypertension Grade 1**	**Hypertension Grade 2**	***P*** **-value for trend**
	**(number = 69)**	**(number = 74)**	**(number = 7)**	**(number = 1)**	
**Kidney Disease**	22 (31.9%)	24 (32.4%)	5 (71.4%)	1 (100%)	0.11
**Microalbuminuria**	16 (23.2%)	21 (28.4%)	5 (71.4%)	1 (100%)	**0.02**
**eGFR <60**	8 (11.6%)	9 (12.2%)	2 (28.6%)	0	0.53
**Among 150 HIV-infected adults on ART**					
	**Normal**	**Prehypertension**	**Hypertension Grade 1**	**Hypertension Grade 2**	***P*** **-value for trend**
	**(number = 49)**	**(number = 58)**	**(number = 33)**	**(number = 10)**	
**Kidney Disease**	14 (28.6%)	19 (32.8%)	22 (66.7%)	7 (70.0%)	**<0.001**
**Microalbuminuria**	13 (26.5%)	16 (27.6%)	22 (66.7%)	7 (70.0%)	**<0.001**
**eGFR <60**	3 (6.1%)	4 (6.9%)	6 (18.2%)	3 (30.0%)	**0.01**

^a^The primary kidney disease outcome: defined as microalbuminuria and/or eGFR <60 mL/min. ART, antiretroviral therapy; eGFR, estimated glomerular filtration rate.

## Discussion

We found that the prevalence of hypertension is high (nearly 30%) among HIV-infected Tanzanian adults on ART for >2 years. In our study, HIV-infected adults on ART had twice the odds of hypertension as HIV-negative controls, even after adjusting for potential confounders, such as age, sex, BMI, waist-hip ratio and vigorous work. HIV-infected adults on ART not only had more hypertension than controls, but also had more severe hypertension (Grade II hypertension – 7% versus 3% of controls). The average blood pressures were not higher among HIV-infected adults on ART, but this is likely because more of the patients in this group were on antihypertensive medications, leading to a reduction in the mean blood pressures. To the best of our knowledge, this is the first study to compare hypertension prevalence between HIV-infected African adults on long-term ART to HIV-negative adults.

The high prevalence of hypertension among HIV-infected adults on ART could be related to dysregulated inflammation due to immune reconstitution. Inflammation is well recognized as a major part of the pathophysiology of hypertension [32]. Activated CD4^+^ T-cells infiltrate the kidney and vascular walls in animal models of hypertension [33]. In our study, among HIV-infected adults, the higher CD4^+^ T-cell counts were associated with more hypertension and higher blood pressures. The prevalence of hypertension was lowest in the group with the lowest average CD4^+^ T-cell count (HIV-infected ART-naive) and highest in the group in which the CD4^+^ T-cell count had been low and had then been reconstituted in the setting of ART. Chronic immune activation, including elevated proportions of activated CD4^+^ and CD8^+^ T-cells and of T-regulatory cells, irreversible loss of gut mucosal integrity and later destruction of lymph nodes, is known to be common among HIV-infected adults [34-36], and may play a key role in the pathophysiology of hypertension among HIV-infected adults on ART in SSA. Chronic inflammation has been shown to persist even after initiation of ART [37]. Even HIV-uninfected adults living in Africa have higher levels of immune activation than their counterparts living in resource-rich settings [38,39], suggesting that hypertension among adults in SSA, and particularly those with HIV infection on ART, may serve as a model for inflammation-induced hypertension in humans. In order to further test this hypothesis, prospective studies are needed to determine the trajectory of blood pressure changes after ART initiation and associated immunologic and inflammatory markers.

Another possible cause of the higher prevalence of hypertension observed among HIV-infected individuals on ART is a direct or indirect effect of the ARV drugs, but we do not think that this is likely to be the primary explanation. In our study, the duration of ART use was not associated with hypertension. Protease inhibitor use was indeed associated with hypertension but only 10% of subjects had received protease inhibitors and other ARV drugs were not associated with hypertension. Moreover, although protease inhibitor use has been associated with hypertension in one prior study [15], most studies showed no association [17,19]. In fact, the majority of studies of hypertension among HIV-infected adults on ART have shown no association between hypertension and ART use, regardless of drug class [12,15-18].

We found that the prevalence of hypertension among HIV-infected, ART-naive adults is low (5%). HIV-infected, ART-naive adults had 65% lower odds of having hypertension when compared to HIV-negative controls, even after controlling for possible confounders, such as age, sex, BMI, waist-to-hip ratio and vigorous work. The lower blood pressures that we observed among HIV-infected, ART-naive adults is consistent with the results of a recent, large meta-analysis that showed that HIV-infected adults in SSA (most not on ART) had lower systolic and diastolic blood pressures than controls [12]. The lower rates of hypertension among HIV-infected, ART-naive adults may be explained by HIV-mediated immunosuppression such that immune reconstitution, after the initiation of ART, causes ‘unmasking’ of hypertension in susceptible individuals. Other explanations, such as dysregulation of the sympathetic nervous system, HIV-related hypoadrenalism and side effects of traditional, herbal medicines, have also been proposed [11,16,17].

We also noted low rates of hypertension diagnosis, treatment and control. Even among HIV-infected adults who were regularly attending the HIV clinic to receive ART, the rates of hypertension awareness, treatment and control were 25%, 15% and 2%, respectively, and only 30% reported undergoing blood pressure measurement in the last year. Similarly low rates of awareness, treatment, control and testing have been described among community-dwelling adults in other parts of SSA [40,41], but one might expect that the situation would be better in the context of ongoing HIV care. Possibly, the low hypertension prevalence observed before ART initiation may be creating a false sense of security with regard to hypertension risk among HIV-infected patients and their care-providers. It is also possible that ART may reduce the effectiveness of anti-hypertensive medications (although this is unlikely to be a major factor in our study since very few of our subjects were taking anti-hypertensive medications at the time of enrollment). On the other hand, HIV care provides a good opportunity for chronic hypertension management [20], and regular blood pressure measurement should be considered an essential element of HIV care as we are now seeking to reinforce in our own HIV clinic. Studies from South Africa have shown that, when non-communicable disease care is integrated into HIV care, HIV-infected adults on ART can attain even better functional ability and health status than the general population [37,42].

Of note, hypertension was also strongly associated with markers of kidney disease in all three study groups; among the 76 total adults with hypertension, 50 (65.8%) had microalbuminuria and 20 (26.3%) had eGFR <60. These findings suggest that the hypertension that we observed in our study is not simply a benign, uncomplicated condition. What remains unclear, however, is whether the hypertension preceded the kidney disease or vice-versa. Kidney disease is known to be common among HIV-infected adults in our region [21] and may be predisposing these adults to the development of hypertension. The kidney disease that we observed among HIV-infected adults on ART does not seem to be related to any specific ART drug based on either this study or our prior work [22].

Study limitations include that enrollment occurred at a single HIV clinic. Our results require validation at other sites. As a government-funded, primary care HIV clinic, though, our patient population is similar to other HIV clinics in our region. In addition, some laboratory tests (such as HIV viral loads) were not available at our center during the study period, but our main study results remain valid even without these variables.

## Conclusions

In conclusion, we observed hypertension in nearly 30% of Tanzanian HIV-infected adults on ART treatment and these adults had twice the odds of hypertension compared to HIV-negative controls, even after correcting for differences in age, sex and adiposity. Among the HIV-infected adults with hypertension, 75% were undiagnosed, 85% were untreated and >95% were uncontrolled. Importantly, hypertension was strongly associated with kidney disease in this population. We suggest that aggressive screening, counseling and treatment for hypertension should be instituted in HIV clinics in SSA. Further studies are needed to determine if this hypertension is due to the ART itself or dysregulated inflammation due to immune reconstitution.

## Abbreviations

ART: antiretroviral therapy
BMC: Bugando Medical Centre
BMI: body mass index
CI: confidence interval
CKD: chronic kidney disease
eGFR: estimated glomerular filtration rate
IQR: interquartile range
JNC-7: Joint National Committee 7
KDIGO: Kidney Disease Improving Global Outcomes
OR: odds ratio
SSA: Sub-Saharan Africa
WHO: World Health Organization
WHR: waist-hip ratio
